# Prevalence of burnout and its association with perceived academic workload among medical undergraduates: A mixed-method cross-sectional study from Mauritius

**DOI:** 10.3126/nje.v16i2.95973

**Published:** 2026-08-03

**Authors:** Indrajit Banerjee, Milakshna Devi Chuckowry, Kshitija Gajadhur, Riya Patil, Arasi Pearia Anadachee, Ranjit Patil

**Affiliations:** 1,4,5,6 Sir Seewoosagur Ramgoolam Medical College, Mauritius; 2,3 Jawaharlal Nehru Hospital, Rose Belle, Mauritius

**Keywords:** Academics, Burnout, Medical students, Predictors, Prevalence

## Abstract

**Background:**

The main objective of this study was to determine the prevalence and academic predictive factors of burnout among medical undergraduate students attending Sir Seewoosagur Ramgoolam Medical College (SSRMC), Mauritius.

**Methods:**

A mixed-methods cross-sectional study was conducted among medical students studying at SSRMC between December 2025 and February 2026.

**Results:**

A total of 232 students participated in the quantitative study with majority being females (147, 63.4%). The Burnout Assessment Tool-Student Version reported an average level of burnout (49.57%) with the exhaustion component being the most significant (mean = 3.45 ± 0.883). Descriptive analysis of the predictive factors showed that academic self-perception had the highest mean (3.266 ± 0.710) and perceptions of workload was identified as the only statistically significant predictor (β = -0.315, t = -3.968, p <0.001). A notable gender disparity was seen with female students demonstrating higher total burnout levels (mean difference 0.29). Qualitative analysis of twenty interviewees focused on identifying the understanding, causes, impact, coping strategies and recommendations for burnout, which revealed tedious academic and examination schedules among the important contributing factors for this phenomenon.

**Conclusion:**

Burnout was highly prevalent among medical students, especially females, with academic workload being the most significant predictor.

## Introduction

Burnout is a psychological syndrome characterized by three dimensions: feelings of emotional exhaustion, depersonalization, and reduced sense of accomplishment; commonly precipitated by mismanagement of chronic workplace stress [1,2]. It is recognized as an occupational phenomenon, particularly common among people-oriented professions, including human services, education and healthcare, negatively impacting their physical and emotional health and resulting in an overall unproductive workplace environment [2]. The definition of burnout was initially restricted to the professional work place; however, recent studies show that burnout is a ubiquitous phenomenon [3].

The demanding and rigorous nature of medical education places medical students at a heightened risk of burnout; affecting almost half of all medical students during their time at university and resulting in an overall global prevalence of 44% [4]. Multiple factors have been identified to explain these high levels: organizational stress due to frequent examinations, increased workload, lack of emotional support during stressful events, inadequate recreation time, highly competitive and hierarchical environment. Medical students tend to be inherently competitive, holding themselves to much higher standards, which, coupled with constant evaluations and scrutiny, leads to increased pressure to perform, especially during clinical years where continuous exposure to sickness and debility results in emotional exhaustion and burnout [5-10].

Studies suggest that at the time of entry into medical schools, those students have mental health parameters comparable to their peers, however, there is a gradual deterioration of their psychological and emotional well-being with time [11]. Burnout affects both the physical and mental health of students, negatively impacting their educational progress by causing fatigue, emotional instability, eating disorders, reduced academic performance and empathy, absenteeism, increased drop-out rates and substance use. The link between burnout and mental health disorders like anxiety, depression, insomnia and suicidal ideation is also well documented [4-9]. Burnout among medical students is of public health importance since long-term consequences of unrecognised burnout may be detrimental to the healthcare system in terms of increased medical errors and negligent behaviours, decreased empathy towards patients, declining ethical and professional standards and overall substandard patient care [5,6]. Detection of burnout at an early stage in one’s career may lead to identification of causative factors and setting up institutional and individual interventions and reforms to tackle this problem, thus improving the educational environment and fostering resilient and empathetic future physicians.

Despite high rates of burnout among medical students in comparable settings, burnout among medical students in Mauritius remains unexplored with no published prevalence studies till date, limiting the development of targeted strategies for its management. This mixed-methods cross-sectional study aims to address this gap by evaluating the prevalence of burnout and its academic predictive factors among undergraduate medical students studying at Sir Seewoosagur Ramgoolam Medical College (SSRMC), Belle-Rive, Mauritius.

An earlier version of this work was presented as a pilot study with a small sample size at the Health Horizon 2025: 5^th^ International Symposium on Medical Sciences held in Mauritius on September 06, 2025.

## Methodology

### Study design and participants

A cross-sectional mixed-method study was conducted among undergraduate medical students attending SSRMC, Mauritius. Study was conducted over a period of 3 months, from December 2025 to February 2026. A total of 232 medical students pursuing MBBS course at SSRMC were taken into consideration for quantitative data. A total of 20 students were included for the qualitative part of the study and were labelled as [S1-S20]. This study adhered to the Strengthening the Reporting of Observational Studies in Epidemiology (STROBE) guidelines [12]. The qualitative report was built upon the Consolidated Criteria for Reporting Qualitative Research (COREQ) [13].

### Inclusion Criteria

Medical students (Semester 2- Semester 10) attending SSRMC during the study period and who were willing to participate in the study were included in this study.

All participants who provided written informed consent before engaging in the project were included.

### Exclusion Criteria

Incomplete questionnaires and student those who were absent were excluded from the study. Since there was no student enrolled in Semester 1, during the time of study period, they were excluded from the study.

### Quantitative data collection

Participants were informed about the study by investigators’ visits to classes. The quantitative questionnaire was distributed via a circulating link on students’ WhatsApp group chats. The survey was a self-administered questionnaire which was distributed via Google Form.

### Questionnaire design

The questionnaire was designed after an extensive literature review. The questionnaire consisted of 3 sections: a.demographic details, b. burnout assessment by Burnout Assessment Tool (BAT) [14] Student Version and c. academic stress assessment by Perception of Academic Stress Scale (PAS) [15]. Demographic details evaluated included gender, age, semester, nationality and current place of residence.

### Questionnaire validity and reliability

Burnout prevalence was assessed using the Burnout Assessment Tool (BAT) Student Version, a pre-validated tool which assesses both core (BAT- C) and secondary (BAT-S) burnout symptoms. The questionnaire has a high internal consistency present for all dimensions assessed. The composite BAT-S, Cronbach’s alpha was 0.90 and for 0.95 for the total BAT-C. [14]. Academic stress was evaluated using the Perception of Academic Stress Scale (PAS), a pre-validated scale with a Cronbach’s alpha internal reliability of 0.7 [15].

### Qualitative data collection

Interview questions were developed based on quantitative data collection results and targeted students’ perceptions of burnout. Questions explored the definition of burnout, experiences of students with burnout, coping strategies, and solutions to burnout.

Interviews were carried out in a confidential one-on-one setting under the supervision of a senior author IB. All interviewers received prior training in qualitative research interviewing methods, followed by a standardized approach to facilitate open and unbiased discussions. Interviews were conducted in a neutral, non-judgmental manner to reduce the potential for interviewer bias. The face-to-face interviews lasted 15-20 minutes. Interviews were manually transcribed by the interviewers.

Qualitative data were coded independtly by two researchers (KG and MDC). Intercoder reliability was evaluated using Cohen’s kappa coefficient, which indicated substantial agreement between the coders (κ=0.80). Coding discepencies were discussed and resolved through consensus, with oversight from the senior author (IB). The final coding framework was established by mutual agreement before proceeding with thematic analysis.

Both quantitative and qualitative data were de-identified to preserve the participants’ privacy. Data collection was carried out by two investigators, AA and RP under the supervision of senior authors, IB and RP(2). Analysts MDC and KG, were blinded and only made aware about the semester, gender and ethnicity of interviewees.

### Outcomes of the study

Primary outcome was the prevalence of burnout and its relation to academic stress among medical sudents.

### Sample size estimation

#### a. Quantitative domain

Sample size was determined prior to data collection using OpenEpi and was estimated to be 220, calculated at the 95% confidence interval [16]. The population size for a finite population was 519, hypothesized frequency of outcome factor in the population was 44.2% and was taken from a meta-analysis assessing the prevalence of burnout among medical students [4]. The confidence limit was set at ± 5% and design effect at 1. To allow better generalisation of results, minimal bias and to enhance the reliability of results, a 5% error margin was allowed, resulting in a final target population of 231.

#### b. Qualitative domain

For the qualitative component, data collection was guided by the principles of Glaser et al. Interviews were continued until thematic saturation was achieved, meaning that no new themes, categories or codes emerged from subsequent interviews [17].

### Statistical analysis

#### a. Quantitative domain

Data were collected and managed using Microsoft Excel 2021 software and analysed using IBM SPSS Statistics 31.0 (SPSS Inc., Chicago, Ill., USA). Descriptive analysis was done, with categorical variables expressed as percentage and included prevalence of each burnout level. Data were further analysed using Pearson correlation, independent sample t-test and multiple regression analysis.

#### b. Qualitative domain

Qualitative data were analysed using NVivo 15 software. An inductive thematic phenomenological analysis was performed for qualitative data analysis. Each transcript was systematically coded, and overarching themes applicable to the study aims were identified.

### Ethics Approval

The study was approved by the institutional research and ethics committee of SSRMC (Approval code: Ref.no. SSRMC/IERB/2025/007) was obtained on September 29, 2025.

### Methodological Triangulation

Methodological triangulation was applied to improve the validity, credibility and depth of this study. The combination of qualitative and quantitative methods enhanced the holistic understanding of burnout by interpreting the findings individually and then, comprehensively.

## Results

### Quantitative Data

A total of 232 responses out of 519 have been recorded with a response rate of 44.7%. The sociodemographic characteristics of the participants are presented in Table 1, where the majority of respondents were female (147, 63.4%). The average age of the medical students was 21.65 ± 1.99 years. The highest proportion of medical students who took part in the study were from semester 4 (16.8%) and the least being in semester 5 (9.1%). Most students were Mauritian (52.2%). The majority of participants resided with their family (53.4%).

As depicted by Table 2, the majority of students experienced an average level of burnout (49.6%), while 24.6%, 21.1% and 4.74% of participants reported low, high and very high levels of burnout, respectively.

The core components of the BAT-SV as shown in table 3 indicates that the exhaustion component had the highest mean (3.45 ± 0.883), followed by cognitive impairment (3.07 ± 0.917), while mental distance had the lowest average (2.72 ±0.871). Additionally, as depicted by table 3, the secondary symptoms experienced by students noted that psychosomatic distress was more common with a mean of 3.31 ± 0.820. Total secondary complaints had an average of 2.83 ± 0.871.

Table 4 depicts the descriptive studies of academic predictive factors of burnout, comprising factors present in the PAS namely, pressures to perform, perception of workload, academic self-perceptions and time restraints. Academic self-perception had the highest mean (3.27 ±0.710), followed by time restraints (2.83 ± 0.651).

Correlation studies between total burnout levels and predictive factors of burnout are presented in Table 5. Pearson correlation showed no statistically significant relationship between age and total burnout (r = -0.006, p = 0.929). Similarly, Spearman’s correlation indicated no significant association between semesters and total burnout (ρ = -0.026, p = 0.690). In contrast, PAS factors showed a statistically significant negative correlation. Perceptions of workload demonstrated a moderate negative correlation (r = -0.576, p<0.001), followed by pressures to perform also showing moderate negative correlation (r = -0.054, p<0.001). Notably, the total PAS score demonstrated the strongest negative correlation (r = -0.607, p<0.001).

Table 6 depicts an independent sample t-test which was conducted to compare total burnout scores between gender groups. There was a statistically significant difference in total burnout between the groups (t = -2.82, p = 0.005). The mean difference was -0.29 (95% CI: -0.50 to -0.09), indicating that burnout levels differed significantly.

Table 7 depicts a multiple regression analysis of multiple variables which predicts total burnout. From the analysis, perceptions of workload emerged as the only statistically significant predictor of total burnout (β = -0.315, t = -3.97, p <0.001). Other academic stressors showed marginal effects which were not statistically significant, such as pressure to perform (β = -0.168, p = 0.0570) and time restraints (β = -0.150, p = 0.0830). Academic self-perceptions (β = -0.0720, p = 0.247) and other sociodemographic factors were not statistically significant predictors. In addition, 39.6% variance was noted in total burnout (R^2^ = 0.396, adjusted R^2^ = 0.377), demonstrating a weak to moderate fit.

### Qualitative Data

A total of 20 students participated in the interviews. The demographics details of the interviewees have been summarised in Table 8.

A thematic analysis was carried out and the following five themes generated: definition of burnout, causes of burnout, impact of burnout, coping strategies and suggestions from students, with the most common codes depicted in Table 9.

Students defined burnout as “...feeling of like I am physically there but mentally, just totally exhausted… my mind is somewhere else…”, with most of them associating burnout with physical and mental exhaustion.

Table 9 illustrates some causes of burnout among the interviewees, with the following codes having the highest frequencies: demanding school timetable (n=15), exam season and studying (n=13), multiple tests on the same day (n=10), exam preparation phase (n=9) and constantly thinking about studies (n=9). Conversely, teachers’ feedback, absent self-satisfaction and having to adapt to a new environment were least frequently observed. Most recurrent codes for impact of burnout were: feeling overwhelmed, burdened or stressed (n=9), reduced academic performance (n=8), sleep disturbances (n=8), self-isolation tendencies (n=7) and work-life imbalance (n=7). The most common coping strategies engaged by participants were: spending time with family (n=12), socialisation (n=9) and sleep or rest (n=6). While most reported coping methods reflected positive coping practices, it was observed that five students employed avoidance coping strategies. Better time management (n=13), increased rest periods or holidays (n=13), revision of the academic timetable (n=6) and improvement of student-teacher relationships (n=5) were the most common suggestions made by students.

### Triangulation of quantitative and qualitative data

The quantitative and qualitative datasets in Table 10 demonstrated exhaustion as one of the most important components of burnout. Perception of workload was also perceived to be a substantial predictor when comparing both data pools. However, interviewees were not in accordance with quantitative findings where pressure to perform was found to be a predictor of burnout. According to interviewees, academic self-perception was a vital predictor of burnout, differing greatly from the quantitative dataset.

## Discussion

### Quantitative Domain

This study assessing the level of burnout and its predictive factors in undergraduate medical students is one of its first in Mauritius. The findings revealed a notable prevalence of burnout across the study population with nearly half of the sample reporting average level of burnout (49.6%). More concerning was that nearly one-quarter of the study population was experiencing clinically significant burnout symptoms with 21.1% having high level and 4.74% having very high level of burnout. This is supported by a study conducted by Rotenstein et al (2018) who noted that the global prevalence of burnout was approximately 44.2% [4].

Beyond prevalence, this study also explored the relationship between burnout and perceived academic stressors namely pressures to perform, perception of workload, academic self-perceptions and time restraints along with sociodemographic factors (gender, age and semesters). The findings indicated that all perceived academic stressors were significantly correlated to burnout with negative correlation coefficients suggesting poorer perceptions of academic conditions. These results reinforced the idea that students’ subjective experiences of their academic environment play an important role in burnout [18,19].

However, demographic variables (age and semesters) were not found to be statistically significant as predictive factors of burnout, indicating that burnout appears to be relatively consistent across different age groups and academic years. This is congruent with a study performed Shrestha DB, et al (2021) where no correlation was found between year of study and burnout level in medical students of Kathmandu despite prior studies showing its correlation [20-21]. Another study by Pleșea-Condratovici C et al (2025) found that younger students tend to experience higher levels of burnout which is contradictory to the findings of the present study [22] . In addition, a significant difference in burnout level between gender was noted. Independent sample t-tests revealed that females had higher burnout level with a mean difference of 0.29, which is in contrast with previous studies where no significant difference was found between gender and burnout [19, 23]. The observed gender difference may also reflect variations in the way burnout symptoms are perceived by different genders.

Furthermore, multiple regression analysis revealed that among all predictors, only perception of workload emerged as a significant independent predictor of burnout. This suggests that workload may be the most influential factor contributing to burnout when other variables are controlled. This is supported by a study conducted by Bruno Coelho Duarte O et al (2025) where academic overload was identified as one of the major risk factors leading to burnout [24].

### Qualitative Domain

Through a thematic qualitative analysis conducted among 20 medical students attending SSRMC, 5 major themes reflecting perceptions of students on burnout were identified, namely: understanding burnout, causes, impact, coping strategies and student-derived recommendations. Students described burnout as feeling mentally and physically exhausted, which was a general consensus among interviewees. These findings are partly consistent with earlier research conducted by Morcos et al (2022), who highlighted that burnout is characterised by emotional exhaustion [5].

The most common causes of burnout identified were having demanding school timetables, examination season and intense studying periods, and having multiple tests on the same day. These were responsible for causing distress among students which in turn led to feelings of burnout. The study by Thun-Hohenstein L et al (2020) was consistent with our findings, demonstrating that burnout symptoms were associated with tight schedules and heavy workload in a 5-year medical program, similar to our study settings [25]. The interviewees also explained that having frequent examinations and intense studying phases contributed to feeling burnout as these periods were often stressful and exhaustive, corroborating the findings of Santiago IS et al (2024) [26]. Additionally, both preclinical and paraclinical students mentioned that multiple tests scheduled on the same day, with minimal or no rest time in between, contributed to feelings of burnout that may be explained by cognitive overload, continuous stress and insufficient recovery time during these periods.

Burnout was found to have a significant impact on participants in the following aspects: cognitive and functional capacity, emotional, academic, lifestyle and social factors. Feeling overwhelmed, burdened or stressed, and changes in lifestyle and social aspects were recurring patterns, as was an increase in arguments with parents, reduced social interaction with peers, sleep disturbances, self-isolation tendencies and work-life imbalance. Findings which were consistent with general studies on the topic [27, 28]. Akiki Z et al (2025) further revealed that social isolation, anxiety or depression have significant associations with poorer quality of life [29].

A multitude of coping strategies were employed in face of burnout with the majority engaging in positive coping practices that included- spending time with family, socialising with friends, sleeping or resting, exercising regularly and listening to music, among others, findings which are congruent with previously published studies [30-32]. Nebhinani N et al (2021) described coping strategies such as substance use, denial or venting while a minority of students in this study reported a form of avoidance coping and explained that when they feel burnout, owing to strict attendance requirements, they instead take a break from studying at home [33].

### Triangulation of Quantitative and Qualitative Domains

Burnout is a multifaceted phenomenon with numerous core dimensions. However, the general consensus of both domains revealed the predominant dimension to be exhaustion which is consistent with earlier findings [5]. This study assessed multiple predictors of burnout out of which only perception of workload emerged as statistically significant from the quantitative data pool which is consistent with the interviewees’ perspectives as well the study conducted by Thun-Hohenstein L et al (2020) [25]. Nonetheless, the findings for pressure to perform as a predictor did not mirror each other. According to interviewees’ perspectives, pressure to perform is one of leading cause of burnout in medical students (Students found pressure to be constant throughout the year and increased even more during exam seasons) but quantitative data did not support this (p= 0.0570). Another conflicting dataset was the academic self-perception data pool where, qualitatively, cause of burnout theme emerged as reduced academic performance “I’m not able to perform to the best of my abilities” which greatly differs with the quantitative dataset (p= 0.247).

### Recommendations

The results of the present study have important implications for students and the academic institution. As Perception of Workload was found to be the strongest predictor of burnout and that females reported higher burnout levels, interventions should focus on reducing academic demands and providing targeted support for vulnerable groups of medical students. Furthermore, interviewees recommended improving time management skills, engaging in regular physical activities and allowing increased rest periods or holidays. Students explained that medical school and their future medical careers are likely to remain hectic and demanding, therefore proactive task initiation and maintenance of well-structured timetables may help prevent burnout. These findings are partially consistent with Abouammoh N et al (2020) which reported that while time management can be seen as stressful for some students, it was still a stress-reliever for others [34].

Students also suggested that having every other Saturday off or providing rest periods following major examinations may contribute to reducing burnout in the long run. The institution could consider the implementation of stress reduction programs through mentorship and encourage self-care practices in students [35,36]. Moreover, early identification of students at high risk of burnout would help prevent more serious outcomes such as psychosomatic distress and complaints and thus, improve the overall well-being and academic performance of students [37].

### Strengths

The primary strength of this study is that it is a mixed-methods cross-sectional study, which triangulates both qualitative and quantitative domains to understand the prevalence of burnout and its academic predictive factors among undergraduate medical students studying at Sir Seewoosagur Ramgoolam Medical College (SSRMC), Mauritius. This study is the first to examine the prevalence of burnout and its predictive factors among medical students in Mauritius, addressing a notable gap in existing literature. Key strengths of the study lie in its relatively large sample size, its use of validated instruments (Burnout Assessment Tool-Student Version, Academic Stress Scale), and the blinding of its analysts to the identity of participants.

### Limitations

This study was a single centre one, and use of self-reported questionnaires may have introduced response bias. Furthermore, burnout levels may vary depending on the timing of data collection, particularly in relation to final board examinations, which could influence stress levels and consequently burnout scores. Although the sample size was adequate, the response rate of this study was 44.7%.

## Conclusion

This study displayed a high prevalence of burnout among medical undergraduates in Mauritius, which was associated with high academic stress, especially among females. Burnout was most significantly associated with the perception of workload that was perceived as positive by most individuals, and reported to be the foundation for internship. Common suggestions to decrease burnout were breaks after exams and more structured class tests and tutorial schedules. Overall, further studies with larger sample sizes in multiple centres need to be carried out to better understand the situation, gender disparity and solutions for alleviating burnout.

### What is known on this topic

• Burnout is emerging as an alarming issue over the recent years, especially in the medical field in higher institutions.

• Rigorous academic schedules, high expectations and competitive culture have led to students experiencing mental and physical exhaustion, stress, along with deterioration of their academic performance.

• The rising significant negative impact of burnout remains a task to eliminate.

### What this study adds

• This study identifies the number of medical students being affected by this phenomenon in a higher institution of Mauritius, helping in recognising the problem at hand.

• The predictive factors of burnout faced by the medical students have also been determined in an attempt to find solutions.

• Recommendations made by students may help policymakers on how to ameliorate the educational system and how to deal with burnout among students.

### Future scope of the study

A multicentric study involving medical students from institutions across Mauritius and including larger sample sizes will enhance the generalizability of findings. Such studies would provide a more comprehensive understanding of the prevalence of burnout and help to identify its academic predictors among medical students across Mauritius.

## Figures and Tables

**Table 1:** Sociodemographic Characteristics (Quantitative Data)

Variables	Number (N=232)	Percentage (%)	95% Confidence Interval [95% CI]
**Gender**			
**Male**	85	36.6	[30.4-42.8]
**Female**	147	63.4	[57.2-69.6]
**Age (years)**			
**<18**	5	2.2	[0.3-4.0]
**18-20**	53	22.8	[17.4-28.2]
**21-22**	96	41.4	[35.0-47.7]
**23-24**	57	24.6	[19.0-30.1]
**>24**	21	9.1	[5.4-12.7]
**Semester**			
**1**	-	-	
**2**	29	12.5	[8.2-16.8]
**3**	25	10.8	[6.8-14.8]
**4**	39	16.8	[12.0-21.6]
**5**	21	9.1	[5.4-12.7]
**6**	22	9.5	[5.7-13.3]
**7**	24	10.3	[6.4-14.3]
**8**	22	9.5	[5.7-13.3]
**9**	28	12.1	[7.9-16.3]
**10**	22	9.5	[5.7-13.3]
**Nationality**			
**Mauritian**	121	52.2	[45.7-58.6]
**Indian**	91	39.2	[32.9-45.5]
**South African**	20	8.6	[5.0-12.2]
**Current Place of Residence**			
**With your family**	124	53.4	[47.0-59.9]
**University Hostel**	47	20.3	[15.1-25.4]
**Rented Accommodation outside of campus**	61	26.3	[20.6-32.0]

**Table 2:** Prevalence of Burnout

Burnout Level	Number (N = 232)	Percentage (%)
**Low**	57	24.6
**Average**	115	49.6
**High**	49	21.1
**Very High**	11	4.74

**Table 3:** Components of Burnout Assessment Tool-Student Version and Total Scoring

Variable	Mean	Standard Deviation	Minimum	Maximum
**Core Components**				
**Exhaustion**	3.45	0.883	1	5
**Mental Distance**	2.72	0.871	1	5
**Cognitive Impairment**	3.07	0.917	1	5
**Emotional Impairment**	2.88	1.0030	1	5
**Secondary Complaints**				
**Psychosomatic Distress**	3.31	0.820	1	5
**Psychosomatic Complaints**	2.72	0.734	1	5
**Burnout Assessment Tool-Student Version Scoring**				
**Total Secondary Complaints**	2.83	0.871	1	5
**Total Burnout**	3.07	0.771	1	5

**Table 4:** Descriptive Studies of Predictive Factors of Burnout

Variables	Mean	Standard Deviation	Minimum	Maximum
**Pressures to perform**	2.746	0.771	1	5
**Perceptions of workload**	2.603	0.857	1	5
**Academic Self-Perceptions**	3.266	0.710	1.4	5
**Time Restraints**	2.834	0.651	1.17	5

**Table 5:** Bivariate Analysis on Total Burnout and Predictive Factors of Burnout

Variable	Correlation (r/ρ)	Test Used	p - value
**Age**	-0.006	Pearson	0.926
**Semesters**	-0.026	Spearman’s	0.690
**Pressures to perform**	-0.554	Pearson	<0.001
**Perceptions of workload**	-0.576	Pearson	<0.001
**Academic Self-Perceptions**	-0.350	Pearson	<0.001
**Time Restraints**	-0.531	Pearson	<0.001
**Total PAS**	-0.607	Pearson	<0.001

**Table 6:** Independent Samples t-test for Gender and Total Burnout

Variable	t	df	p- value	Mean Difference	95% Confidence IntervalLower	95% Confidence Interval Upper
**Total Burnout**	-2.82	230	0.005	-0.29	-0.50	-0.09

**Table 7:** Multiple Regression Analysis Predicting Total Burnout

Predictor	B	SE	β	t	p- value
**Constant**	4.72	0.287	-	16.5	<0.001
**Pressure to Perform**	-0.168	0.0880	-0.168	-1.92	0.0570
**Perceptions of workload**	-0.284	0.0710	-0.315	-3.97	<0.001
**Academic Self- Perceptions**	-0.078	0.0680	-0.0720	-1.16	0.247
**Time Restraints**	-0.178	0.102	-0.150	-1.74	0.0830
**Gender**	0.130	0.0840	0.0820	1.543	0.124
**Age**	0.0520	0.0560	0.0650	0.927	0.355
**Semesters**	-0.0100	0.0200	-0.0320	-0.467	0.641

**Table 8:** Demographic Details (Qualitative Data)

		Frequency (n)	Percentage (%)
**Gender**	**Female**	11	55.0
	**Male**	9	45.0
**Nationality**	**Indian**	3	15.0
	**Mauritian**	14	70.0
	**South African**	3	15.0
**Study Phase**	**Preclinical**	3	15.0
	**Paraclinical**	6	30.0
	**Clinical**	11	55.0

**Table 9:** Thematic Phenomenological Analysis

Themes	Codes	Description	Cases (Number of participants)	Counts (Number of Evidence)	Evidence
**Causes of Burnout**	Demanding School Timetable	Densely packed school schedule with minimal to no breaks in between	15	39	“and also how are timetable is framed...you finish at 4 everyday and then we have school on Saturdays as well” [S8]
	Exam Season and Studying	Period of studying before and during major exams	13	23	“....when I entered this college, pressure is throughout the year but more intense during exam season” [S4] “Over the exams....gradually as the exams progress i get more burnout....you know when you have go through that stress for a long period of time” [S8]
**Impact of Burnout**	Feeling overwhelmed, burdened or stressed	Emotional state linked to burnout	9	9	“the environment in this college...there is too much pressure...I just feel that everyone is just so stressed all the time, and that makes me feel stressed even when I’m not” [S12] “overwhelmed by the workload” [S15]
	Reduced academic performance	Poor performance in studies	8	13	“I’m not able to perform to the best of my abilities” [S6] “Burnout definitely decreases my ability to perform…even things which I know, I am not able to reproduce them” [S5]
**Coping Strategies**	Spending time with family	Engagement in familial moments and activities	12	16	“I talk to my mom and that helps to bring me back to baseline” [S9] “Family and friends support is the main thing...” [S16]
	Socialisation	Act of conversing with others	9	11	“I try to maintain a balanced social life and socialise.” [S10]
**Suggestions from students**	Better time management	Skills in efficiently allocating time for tasks	13	20	“Better time management on the part of the students..” [S3] “not keeping everything for the last minute and having a balance in the beginning itself helps, and helps prevent me from burnout” [S9]
	Increased rest periods or holidays	Promotion of longer leave periods or vacations	13	15	“I would say maybe having a weekend off every month or having at least 1 week off after semester so students coming from outside can go visit their family a bit more often, so that we can decompress better and start the next semester with a clearer head. “ [S7]

**Table 10:** Data Triangulation

Quantitative factor (Mean/ p value)	Qualitative theme	Relationship between the two
**Exhaustion component (Mean =3.45)**	Feeling overwhelmed, burdened or stressed “overwhelmed by the workload”[S15]	Both findings are in accordance with each other
**Perception of workload (p<0.001)**	Demanding school timetable: Tight schedule of classes “and also how are timetable is framed...”[S8]	The interviews corroborate with the findings
**Pressure to Perform (p=0.0570)**	Period of studying before and during major exams “....when I entered this college, pressure is throughout the year but more intense during exam season” [S4]	Interviewees view pressure to perform as a major predictor despite findings being statistically not significant
**Academic Self- Perceptions (p=0.247)**	Reduced academic performance “I’m not able to perform to the best of my abilities” [S6]	According to interviewees, academic self perception is a vital predictor of burnout differing greatly from the quantitative dataset
